# Identification and Validation of New Cancer Stem Cell-Related Genes and Their Regulatory microRNAs in Colorectal Cancerogenesis

**DOI:** 10.3390/biomedicines9020179

**Published:** 2021-02-11

**Authors:** Kristian Urh, Margareta Žlajpah, Nina Zidar, Emanuela Boštjančič

**Affiliations:** Faculty of Medicine, Institute of Pathology, University of Ljubljana, 1000 Ljubljana, Slovenia; kristian.urh@mf.uni-lj.si (K.U.); margareta.zlajpah@mf.uni-lj.si (M.Ž.); nina.zidar@mf.uni-lj.si (N.Z.)

**Keywords:** colorectal cancer, differentially expressed genes, cancer stem cells, qPCR

## Abstract

Significant progress has been made in the last decade in our understanding of the pathogenetic mechanisms of colorectal cancer (CRC). Cancer stem cells (CSC) have gained much attention and are now believed to play a crucial role in the pathogenesis of various cancers, including CRC. In the current study, we validated gene expression of four genes related to CSC, *L1TD1*, *SLITRK6*, *ST6GALNAC1* and *TCEA3*, identified in a previous bioinformatics analysis. Using bioinformatics, potential miRNA-target gene correlations were prioritized. In total, 70 formalin-fixed paraffin-embedded biopsy samples from 47 patients with adenoma, adenoma with early carcinoma and CRC without and with lymph node metastases were included. The expression of selected genes and microRNAs (miRNAs) was evaluated using quantitative PCR. Differential expression of all investigated genes and four of six prioritized miRNAs (*hsa-miR-199a-3p*, *hsa-miR-335-5p*, *hsa-miR-425-5p*, *hsa-miR-1225-3p*, *hsa-miR-1233-3p* and *hsa-miR-1303*) was found in at least one group of CRC cancerogenesis. *L1TD1*, *SLITRK6*, *miR-1233-3p* and *miR-1225-3p* were correlated to the level of malignancy. A negative correlation between *miR-199a-3p* and its predicted target *SLITRK6* was observed, showing potential for further experimental validation in CRC. Our results provide further evidence that CSC-related genes and their regulatory miRNAs are involved in CRC development and progression and suggest that some them, particularly *miR-199a-3p* and its *SLITRK6* target gene, are promising for further validation in CRC.

## 1. Introduction

Colorectal cancer (CRC) is ranked as the third most common cause of morbidity due to cancer worldwide [1]. The five-year survival of patients with CRC can vary, with five-year survival rates of approximately 90% in patients with adenoma with early carcinoma and approximately 8–12% in patients with advanced CRC [2]. Despite the introduction of new treatment modalities, 40–50% of CRC patients develop metastases [1,2,3,4]. The prognosis can be improved significantly with the detection of early lesions through population screening programs [5,6].

CRC development is divided into discrete stages, ranging from normal mucosa to invasive carcinoma. The majority of CRC cases develop from precursor lesions, adenomas and serrated polyps [4]. Molecular pathways involved in CRC development include stepwise accumulation of mutations, epigenetic changes, and changes in gene expression, leading to uncontrolled cell division and an invasive phenotype [4,7]. Most genetic events that are associated with tumour development occur early, before the formation of the adenoma, leading to an urgent need to define mechanisms responsible for the switch from adenoma to carcinoma.

It is believed that the bulk of any given neoplasm consists of cells incapable of metastatic seeding or tumour progression. A minority of cancer cells, referred to as cancer stem cells (CSC) or CSC-like cells [8], are capable of self-renewal, differentiation and mobility. They are mostly found as a subpopulation on the invasive tumour front, and are believed to be responsible for invasiveness, metastatic spread and relapse [9,10]. Additionally, turnover of CSCs is slow, which in turn allows greater resistance to therapies that target rapidly replicating cells [9,10]. 

Two separate mechanisms have been suggested for the development of CSC in CRC. According to the first, oncogenic mutations accumulate within the colonic crypt stem cells, located in the bottom area of a normal crypt. These CSCs are able to differentiate into mature cancer cells and exhibit uncontrolled proliferation. According to the second mechanism, cancer cells undergoing an accumulation of genetic changes and/or epithelial-mesenchymal transition dedifferentiate from normal mature epithelial cells into a state similar to stem cells [11]. 

In a previous study, we used a bioinformatics analysis of publicly available gene expression microarray projects [12] and identified potential markers for differentiation between normal colon mucosa, adenoma and CRC. Some of the differentially expressed genes were associated with CSC-like cells, namely *L1TD1*, *SLITRK6*, *ST6GALNAC1* and *TCEA3. L1TD1,* a gene-encoding RNA-binding protein, has been identified as a marker for human embryonic stem cells, their renewal and cancer cell proliferation. It has been associated with RNA transcription, splicing, processing, localization, stability and translation [13,14,15,16]. *SLITRK6*, an integral membrane protein, has been found to be highly expressed in human adult neural stem-like cells and in several cancers. It has been associated with cell adhesion and actin cytoskeleton [17,18,19], cell features that are closely related to cell differentiation, stemness, cancer cell migration and invasion [20,21,22]. *ST6GALNAC1*, encoding an enzyme, has been associated with cell migration, contact and maintenance of isolated CRC stem cells. It is involved in the activation of akt pathway and it is a potential candidate for CSC targeting therapy [23]. *TCEA3*, a transcription elongation factor, has been shown to regulate differentiation of mouse embryonal stem cells through the Lefty1-Nodal-Smad2 pathway [24]. 

However, there is very limited information about their role in CRC. We therefore analysed the expression of these four genes during CRC cancerogenesis, from normal mucosa, adenoma and adenoma with early carcinoma to advanced CRC, predicted miRNAs that could regulate these genes and analysed their expression as well.

## 2. Materials and Methods

### 2.1. Patient and Tissue Selection

Patients who underwent excision or resection of adenoma, adenoma with early carcinoma and CRC from 2015 to 2019 were included in the study. For routine histopathologic examination, tissue samples were fixed in 10% buffered formalin and embedded in paraffin (FFPE). During routine examination, all specimens were evaluated by a pathologist according to standard procedures and, after histopathologic examination, pTNM (pathologic Tumour Node Metastasis) classification was assessed on the basis of the depth of invasion and extent of the primary tumour, the number of lymph nodes with metastases and the presence of distant metastases (AJCC 8th edition [25]). For the purpose of this study, biopsy samples were collected retrospectively from the archives of the Institute of Pathology, Faculty of Medicine, University of Ljubljana. After re-evaluation of consecutive cases for each group by a pathologist and initial quality check, representative samples were selected for further study. Samples of normal mucosa obtained from resected CRC specimens were used as control samples. Patients treated by radiotherapy, chemotherapy or biologic drugs prior to surgery were not included in this study. Patients with mucinous carcinomas or signet cell carcinomas were also excluded. Only sporadic CRC cases were included. Tissue samples were grouped as normal mucosa, adenoma, adenoma with early carcinoma, CRC without lymph node metastases (CRC N0) or CRC with lymph node metastases (CRC N+). 

The study was conducted according to the guidelines of the Declaration of Helsinki, and approved by the National Medical Ethics Committee (Republic of Slovenia, Ministry of Health), approval number 0120-54/2020/4.

### 2.2. Target miRNAs Identification and Prioritization

For the identified differentially expressed genes (DEGs), we searched for miRNA targets that might be involved in the regulation of their expression. The databases MiRTar [26], miRDB [27], Mirna-coadread [28], TarBase [29], TargetScan [30], miRBase [31] and a literature based search on Pubmed, as well as the settings used in the miRNA mining, are given in Table S1.

miRNAs that could target selected DEGs were checked in the miRBase [31] for annotation, method of identification and validation. Cases in which the miRNAs were identified as not true miRNAs were discarded. Only miRNAs either with a known functional association with cancer or that appeared in at least two databases as related to the target gene were considered as potential regulators of DEGs. The identified miRNAs from Table S1 were further prioritized as explained below. 

Alignment between the miRNA and gene sequence was inspected manually and mismatches in the seed region were noted. In cases in which there was a maximum of one mismatch in the miRNA seed binding region in the binding relevant 2–7 bp, the matching was considered sufficient for further analysis [32]. Additionally, in cases in which a relevant reference for cancer associations was identified, the miRNA was also considered for further analysis. We identified the sequence 70 bp and 30 bp upstream and downstream of the mature miRNA binding site, the former for minimum free energy (ΔG) determination in regard to the folding of the sequence and 30 bp for secondary structure analysis [33,34]. Higher ΔG upstream or downstream of the binding site may imply binding issues, whereas a lower ΔG suggests a locally linear RNA structure around the target mRNA-binding site [34]. We also identified ΔG of the potential binding site and identified cases in which the difference between the potential binding site and the 70 bp flanking 3′ and 5′ was at least 10 kcal/mol [35]. ΔG and secondary structure analysis was performed using mFold [36] and Vienna RNAfold [37]. Identification of secondary structures and destabilising elements (DSE) or stabilising elements (SE) was performed for each miRNA-binding site and the 30 bp flanking sequence on each side. Potential DSEs with the following cut-off lengths include a hairpin loop, ≥11 bp; interior loop, ≥9 bp; bulge loop, ≥7 bp; multiple branching loop ≥ 11 bp; and joint sequence or free end, ≥11 bp. DSE could aid in miRNA binding while stabilising elements (SE) including stems, as explained by Zhao, Samal and Srivastava [34]. Structures were considered significant for inhibition of miRNA binding if the ΔG of the structure was lower than −6 kcal/mol [38]. Identification of potential conservation of miRNA-target gene binding site sequences between human, mouse, rat and chicken was performed using TargetScan 7.2 [30]. 

**Figure 1:**
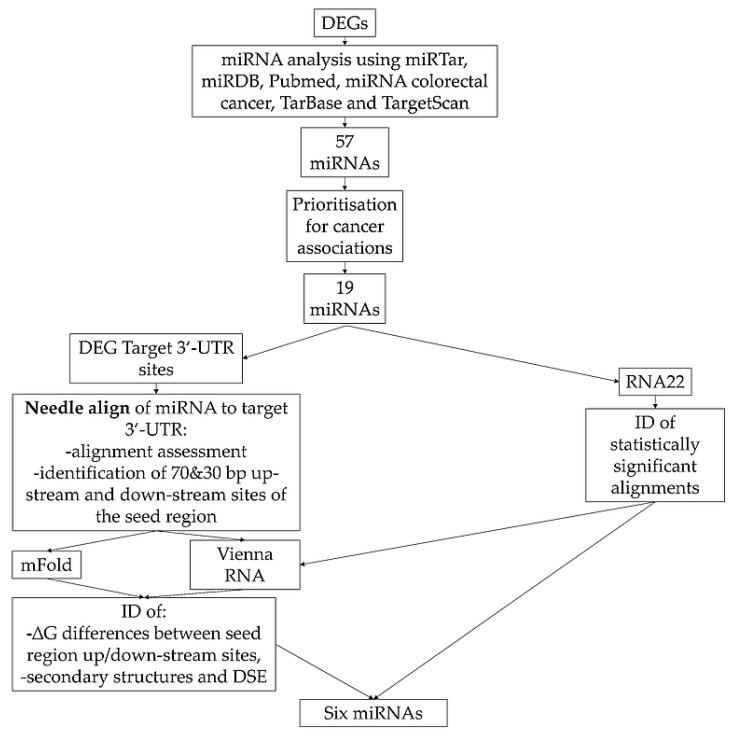
Identification and prioritization of miRNAs. Legend: DEG, differentially expressed genes; DSE, destabilising elements; ΔG, minimum free energy; ID, identification.

RNA22 [39] was used for identification of statistically significant (*p* ≤ 0.05) alignments between DEG target 3′-UTR sites and miRNAs. The settings used in the analysis were: 8-mer or 7-mer seed binding, 1 unpaired sequence in seed region, 1 G:U wobble, maximum folding energy for heteroduplex −12.0 and 20.0 kcal/mol. The heteroduplex energies with cut-off −12.0 and −20.0 kcal/mol used in RNA22 were the energies suggested by the software and the typical setting described in the study by Miranda, Huynh, Tay, Ang, Tam, Thomson, Lim and Rigoutsos [39]. Results of individual analyses were compared for possible overlaps. The full workflow of the prioritization is shown in Figure 1.

### 2.3. RNA Isolation and Quality Assessment

RNA was obtained from FFPE tissue slides using a microtome (4 × 10 µm-thick slides). RNA, including miRNAs, was isolated using an AllPrep DNA/RNA FFPE kit (Qiagen, Hilden, Germany) kit according to the manufacturer’s protocol. Concentration and quality assessment of the isolated RNA was performed using a spectrophotometer ND-1000 or ND-One (Nanodrop, Thermo Fisher Scientific, Waltham, MA, USA) at wavelengths 260 nm and 280 nm. Prior to further analysis, RNA quality was tested using reverse transcription and amplification of *GAPDH* (Hs_GAPDH_vb.1_SG, 100 bp) by SybrGreen technology. Samples that did not amplify during this initial control step were excluded from further analysis.

### 2.4. Reverse Transcription (RT) and Pre-Amplification

Reverse transcription (RT) of the isolated mRNA was performed using One*Taq^®®^* RT-PCR Kit (New England Biolabs, Ipswich, MA, USA) using a mix of random hexamers and oligo-dT primers according to the manufacturer’s protocol. We used 60 ng of RNA in the total 10 µL RT reaction and 1 µL of random hexamers, and incubated for 5 min at 70 °C. Afterward, we added 5 µL of the Reaction mix and 1 µL of Enzyme mix to the reaction and incubated at 25 °C for 5 min, 42 °C for 1 h and 80 °C for 5 min. 

Preamplification of the obtained cDNA was performed using the TaqMan*^®®^* Preamp Master Mix (Thermo Fisher Scientific, Waltham, MA, USA) according to the manufacturer’s instructions. For a 10 µL reaction, we added 5 µL of PreAmp Master Mix (2×), 2.5 µL of Pooled TaqMan^®®^ Gene Expression probes (Thermo Fisher Scientific, Waltham, MA, USA) (0.2×, diluted in TE buffer) and 2.5 µL of cDNA. Incubation was performed at 95 °C for 10 min, 95 °C for 15 s and 60 °C for 4 min.

RT of the isolated miRNAs was performed using the TaqMan™ MicroRNA Reverse Transcription Kit (Applied Biosystems, Foster City, CA, USA) according to the manufacturer’s protocol. The reaction volume was a total of 10 µL, including 10 ng of RNA, 2 µL of RT primer, 0.1 µL of 100 mM dNTPs, 1 µL of MultiScribe™ Reverse Transcriptase 50 U/µL, 1 µL of 10× Reverse Transcription Buffer, 0.19 µL of the RNase Inhibitor 20 U/µL and 0.71 µL nuclease-free water. The conditions for the reverse transcription were 30 min at 16 °C, 30 min at 42 °C and 5 min at 85 °C.

### 2.5. Selection of Primers and Probes

The TaqMan-based approach (Thermo Fisher Scientific, Waltham, MA, USA) was used for the quantitative real-time PCR (qPCR) methodology. A predesigned mixture of primers and probes was used for expression analysis of mRNAs of DEGs and their potential regulatory miRNAs relative to reference genes (RGs). The candidate genes were selected after a bioinformatics analysis performed in a previous study [12]. The potential regulatory miRNAs were selected as described above. Selected probes are shown in Table 1, with reference genes (RGs) presented in bold.

### 2.6. Quantitative Real-Time PCR (qPCR)

Prior to qPCR amplification, efficiencies were determined in triplicate reactions for each probe and for each group of samples. The dilution series included 4-point dilutions ranging from 5-fold to 625-fold for mRNAs/miRNAs. A Rotor Gene Q (Qiagen, Hilden, Germany) machine was used for all qPCR analyses, and all 10 µL testing reactions were performed in duplicate. For mRNAs, the cycling protocol was 50 °C for 2 min, 95 °C for 10 min, 40 cycles of 95 °C for 15 s and 62 °C for 1 min. For miRNAs, the cycling protocol was 95 °C for 10 min, 40 cycles of 95 °C for 15 s and 60 °C for 60 s. The reactions included 5.0 µL of the FastStart™ PCR Master mix (Roche Diagnostics, Basel, Switzerland), 0.5 µL of the TaqMan probe and 4.5 µL of cDNA (pre-amplified cDNA diluted 5-fold for mRNAs and for miRNAs cDNA diluted 100–fold). 

After efficiency correction, the obtained ΔCq (normalized Cq of analysed mRNAs/miRNAs relative to geometric mean of RGs) were used for analysis of target gene/miRNA expression. The fold difference in the expression was calculated against the normal mucosa samples group using the ΔΔCq method [40].

### 2.7. Statistics

Differences in expression were compared between tumour and corresponding normal mucosa using ΔCq and the Willcoxon Rank test (nonparametric test for dependent samples). For comparison of relative quantification of mRNAs/miRNAs between independent groups of samples (e.g., adenoma vs. normal mucosa), ΔCq and the Mann–Whitney U test were used (nonparametric test for independent group of samples). ΔΔCq and the Mann–Whitney U test were used for comparison between CRC N0 and CRC N+ sample groups. Using the Spearman coefficient, we analysed whether miRNAs and the target mRNA were in reverse correlation and whether miRNAs and mRNAs were associated with cancerogenesis. All statistical analyses of experimental data were performed using SPSS version 24 (SPSS Inc., Chicago, IL, USA). Differences in expression between groups were considered significant at *p* ≤ 0.05.

## 3. Results

### 3.1. Patient Characteristics

Approximately 30% of retrospectively selected cases successfully passed initial quality control. Our study therefore included 70 biopsy samples from 47 patients with adenoma (*n* = 11), adenoma with early carcinoma (*n* = 13), CRC without lymph node metastases (*n* = 10) and CRC with lymph node metastases (*n* = 13). There were 15 women and 32 men, aged 73.7 ± 8.4 and 65.7 ± 11.4 years, respectively. As a control group, microscopically normal mucosa from CRC resected specimens was used (*n* = 23). Demographic characteristics of the included patients are shown in Table 2.

Among adenomas, there were six cases of tubular adenoma with high-grade dysplasia, three tubulovillous adenomas with high-grade dysplasia and two tubulovillous adenomas with low-grade dysplasia. Among adenomas with early carcinoma, there were six tubulovillous adenomas, six tubular adenomas and one villous adenoma, all with high grade dysplasia and with malignant transformation, evidenced by invasion of the dysplastic glands in the submucosa (pT1). Among CRC cases, there were two stage I carcinomas, five stage IIA, two stage IIB, eight stage IIIB, one stage IIIC, four stage IVA and one stage IVB carcinomas. Of the CRC cases, 7 cases were poorly differentiated and 16 were moderately differentiated. 

### 3.2. Differential Gene Expression

#### 3.2.1. Differential Gene Expression in Adenoma and Adenoma with Early Carcinoma

**Figure 2:**
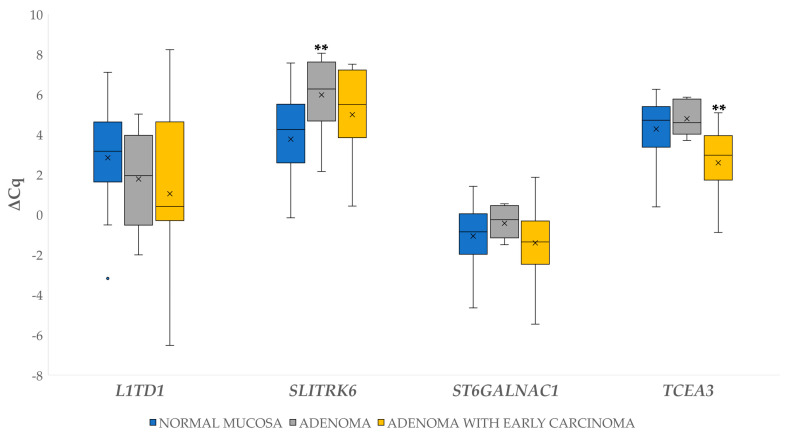
Expression (∆Cq) of four genes (*L1TD1, SLITRK6, ST6GALNAC1, TCEA3)* in normal mucosa, adenoma and adenoma with early carcinoma. Legend: x, mean; ◦, outlier; ** *p* ≤ 0.01.

∆Cq for the investigated genes in adenoma and adenoma with early carcinoma were statistically evaluated independently against normal mucosa samples. Statistically significant results include 6.20-fold downregulation of *SLITRK6* (*p* = 0.010) in adenomas, and 3.22-fold upregulation of *TCEA3* in adenomas with early carcinoma (*p* = 0.006). The results are shown in Figure 2. 

We also observed statistically significant 4.58-fold upregulation in adenoma with early carcinoma compared to adenoma for the gene *TCEA3* (*p* ≤ 0.001).

#### 3.2.2. Differential Gene Expression in Carcinoma Compared to Normal Mucosa

**Figure 3:**
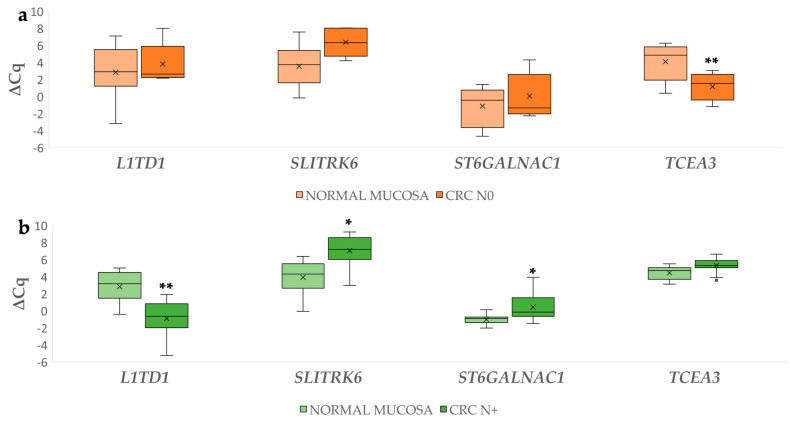
Expression (∆Cq) of the investigated genes in carcinoma without (**a**) and with lymph node metastases (**b**) and corresponding normal mucosa. Legend: CRC N0, colorectal carcinoma without lymph node metastases; CRC N+, colorectal carcinoma with lymph node metastases; x, mean; ◦, outlier; * *p* ≤ 0.05; ** *p* ≤ 0.01.

Differences in expression of the investigated genes between CRC N0 or CRC N+ and corresponding normal mucosa were calculated using ∆Cq. Statistically significant results include the 7.16-fold upregulation of *L1TD1* in CRC N+ (*p* = 0.008) and 6.16-fold downregulation of *SLITRK6* (*p* = 0.039) and 3.10-fold for *ST6GALNAC1* (*p* = 0.02) in CRC N+. Additionally, 7.97-fold upregulation of *TCEA3* in the CRC N0 (*p* = 0.004) was also observed. The results are shown in Figure 3.

#### 3.2.3. Gene Expression in Carcinoma with Lymph Node Metastases Compared to Carcinoma without Lymph Node Metastases

**Figure 4:**
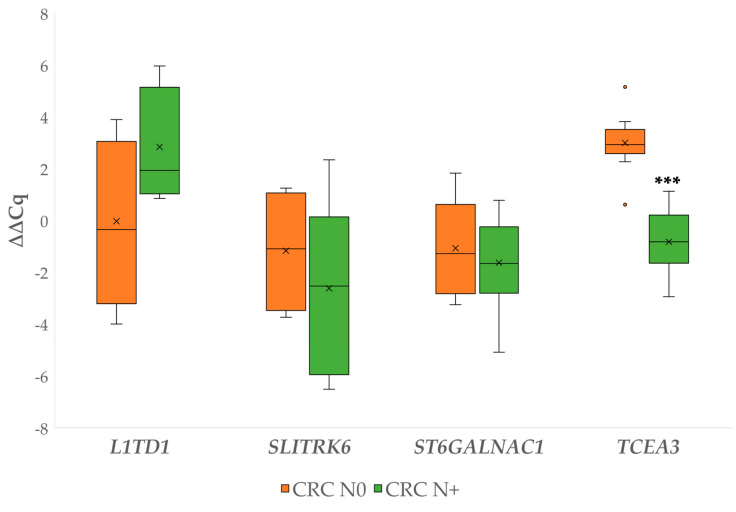
Expression (∆∆Cq) of the investigated genes (*L1TD1*, *SLITRK6*, *ST6GALNAC1*, *TCEA3*) in carcinoma without and carcinoma with lymph node metastases. Legend: CRC N0, colorectal carcinoma without lymph node metastases; CRC N+, colorectal carcinoma with lymph node metastases; x, mean; ◦, outlier; *** *p* ≤ 0.001.

∆Cq values for each carcinoma case were first calculated against the corresponding normal mucosa. Then, the independent ∆∆Cq comparisons for the investigated genes between the CRC N0 and CRC N+ were performed. Statistical significance was identified for *TCEA3*, which was upregulated in the CRC N0 group compared to CRC N+ (*p* ≤ 0.000). The results are shown in Figure 4. The complete statistical comparisons are available in Table S2.

### 3.3. Prioritization of Potential miRNA-Target Gene Associations

Only miRNAs with a known functional association with cancer or which appeared in at least two databases in correlation with the target gene were considered for further prioritization. 

The complete results of the miRNA prioritization for target genes correlations with relevant information and manual alignment with free energy comparisons and secondary structure identification are available in Tables S3 and S4. The complete results of the RNA22 analysis are presented in Table S5.

After comparison of the results of analyses presented in Tables S3–S5, we identified several miRNAs for further validation. A condensed view of choosing a specific miRNA for further validation in association with a potential target gene is presented in Table 3. The minimum requirements are in bold. Only cases with DSEs present in the sequence, a known previous association with the target gene and a previous association with CRC, are included.

### 3.4. Differential miRNA Expression

#### 3.4.1. Differential Expression of miRNAs in Adenoma and Adenoma with Early Carcinoma

We compared ∆Cq values of the investigated miRNAs in adenomas and adenoma with early carcinoma to normal mucosa samples. Among the investigated miRNAs, *miR-335-5p* was not expressed in normal mucosa, adenoma and adenoma with early carcinoma.

**Figure 5:**
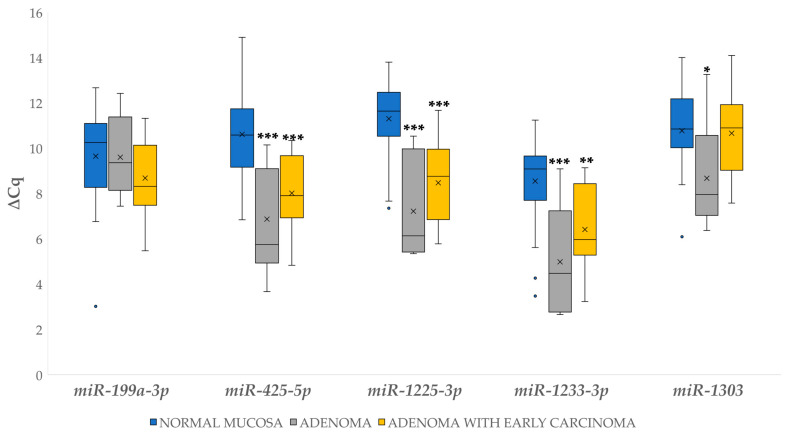
Expression (∆Cq) of the investigated miRNAs in normal mucosa, adenoma and adenoma with early carcinoma. Legend: x, mean; ◦, outlier; * *p* ≤ 0.05; ** *p* ≤ 0.01; *** *p* ≤ 0.001.

Statistically significant changes in expression included upregulation for the majority of miRNAs in both adenoma and adenoma, with early carcinoma in comparison to normal mucosa: 13.43-fold and 6.07-fold for *miR-425-5p* (*p* < 0.001, *p* < 0.001), respectively; 16.97-fold and 6.78-fold for *miR-1225-3p* (*p* < 0.001, *p* < 0.001), respectively; and 11.86-fold and 4.40-fold for *miR-1233-3p* (*p* < 0.001, *p* = 0.003), respectively. *miR-1303* was significantly 4.29-fold upregulated only in the adenoma group (*p* = 0.025). The results are shown in Figure 5.

#### 3.4.2. Differential miRNA Expression in Carcinoma with and without Lymph Node Metastases Compared to Corresponding Normal Mucosa

**Figure 6:**
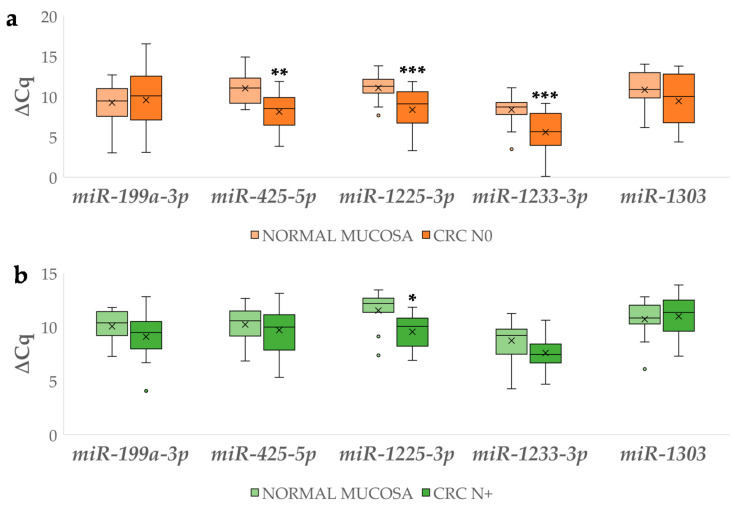
Expression (∆Cq) of the investigated miRNAs in carcinoma without (**a**) and with lymph node metastases (**b**) in comparison to corresponding normal mucosa. Legend: CRC N0, colorectal carcinoma without lymph node metastases; CRC N+, colorectal carcinoma with lymph node metastases; x, mean; ◦, outlier; * *p* ≤ 0.05, ** *p* ≤ 0.01; *** *p* ≤ 0.001.

∆Cq values for the investigated miRNAs were compared between CRC N0 and CRC N+ and their corresponding normal mucosa, as shown in Figure 6. Statistically significant results include the 7.38-fold upregulation of *miR-425-5p* (*p* = 0.002), 6.60-fold for *miR-1225-3p* (*p* = 0.001) and 6.95-fold for *miR-1233-3p* (*p* = 0.001) in CRC N0 and 3.28-fold for *miR-1225-3p* (*p* = 0.019) in CRC N+. Among the investigated miRNAs, *miR-335-5p* was expressed neither in normal mucosa nor in CRC.

#### 3.4.3. Differential Expression of miRNAs Between Carcinoma with and without Lymph Node Metastases

**Figure 7:**
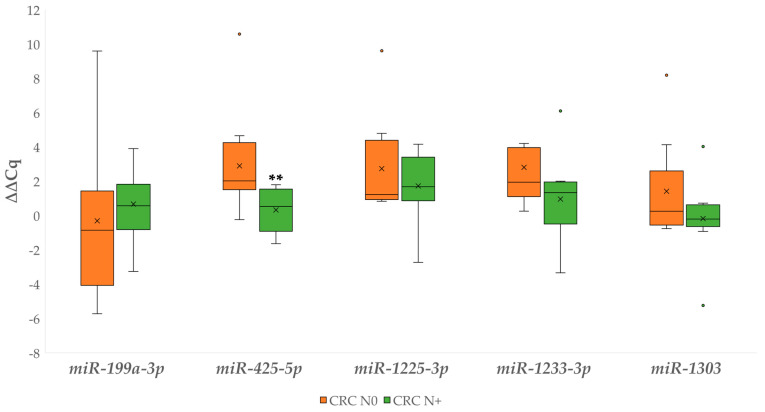
Expression (∆∆Cq) of the investigated miRNAs in carcinoma without and with lymph node metastases. Legend: CRC N0, colorectal carcinoma without lymph node metastases; CRC N+, colorectal carcinoma with lymph node metastases; x, mean; ◦, outlier; ** *p* ≤ 0.01.

Figure 7 shows independent ∆∆Cq comparisons for the investigated miRNAs between the CRC N0 and CRC N+, which revealed a statistically significant difference in the expression of *miR-425-5p* (*p* = 0.003). Additional statistical comparisons are available in Table S6.

### 3.5. Correlation between Expression of Investigated Genes and Their Potentially Regulatory miRNAs

**Figure 8:**
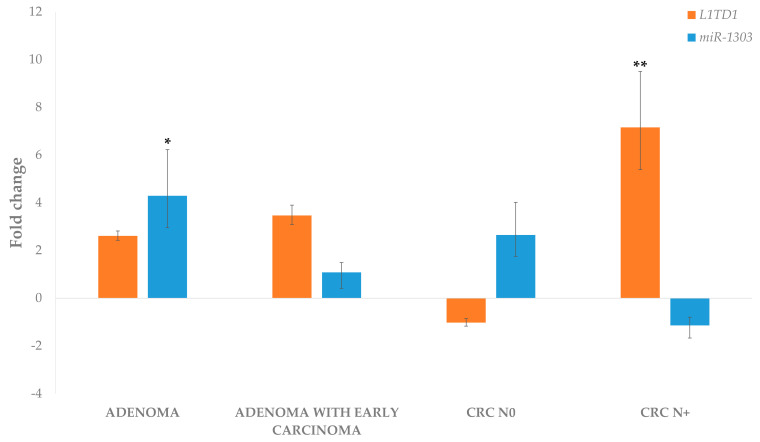
Expression of *L1TD1* and *miR-*1303 in adenoma, adenoma with early carcinoma and carcinoma without and with lymph node metastases. Legend: CRC N0, colorectal carcinoma without lymph node metastases; CRC N+, colorectal carcinoma with lymph node metastases; * *p* ≤ 0.05; ** *p* ≤ 0.01.

The expression of *L1TD1* to *miR-1303*, as shown in Figure 8, showed an inverse trend in all analysed groups except the adenoma group. However, we were not able to confirm a negative correlation between *L1TD1* and *miR-1303* (Table 4).

**Figure 9:**
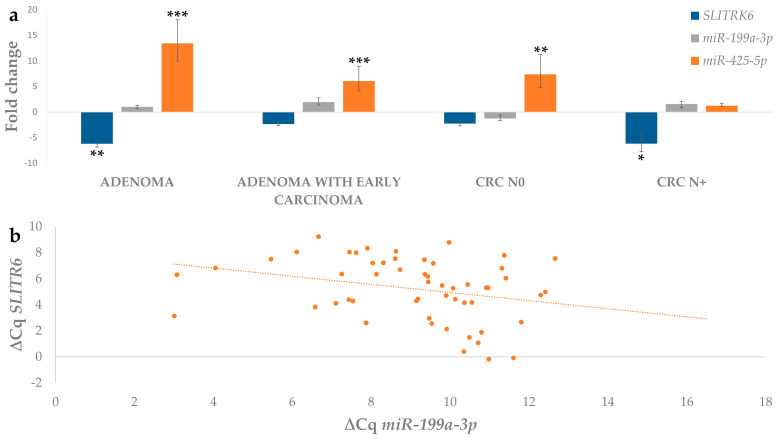
(**a**) Expression of *SLITRK6*, *miR-199a-3p* and *miR-425-*5p in adenoma, adenoma with early carcinoma and carcinoma without and with lymph node metastases; (**b**) Correlation between expression (∆Cq) of *miR-199a-3p* and target gene *SLITRK6*. Legend: CRC N0, colorectal carcinoma without lymph node metastases; CRC N+, colorectal carcinoma with lymph node metastases; * *p* ≤ 0.05; ** *p* ≤ 0.01; *** *p* ≤ 0.001.

Comparing the fold change expression data for *SLITRK6* with the predicted miRNAs *miR-425-5p* and *miR-199a-3p,* we observed an inverse trend of expression between *miR-425-5p* and *SLITRK6* in all tested groups. Expression of *miR-199a-3p* remained at similar levels throughout the adenoma-carcinoma progression. The results are shown in Figure 9a. However, we were able to confirm a negative correlation between *SLITRK6* and *miR-199a-3p,* as shown in Figure 9b. The correlation testing results are given in Table 4.

**Figure 10:**
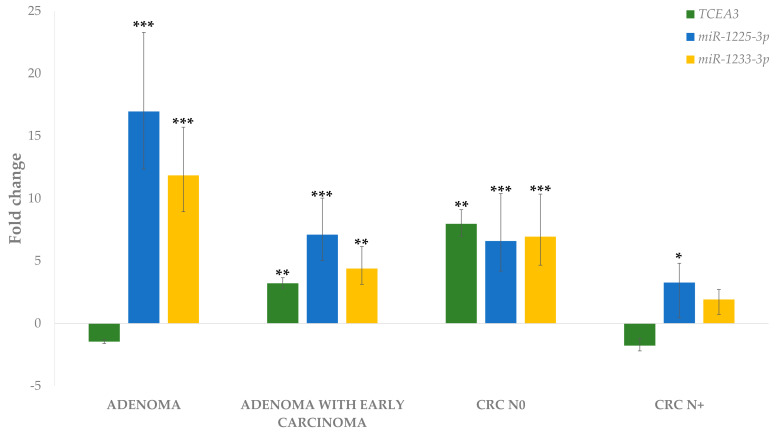
Expression of *TCEA3*, *miR-1225-3p* and *miR-1233-3p* in adenoma, adenoma with early carcinoma and carcinoma without and with lymph node metastases. Legend: CRC, colorectal carcinoma; N0, without lymph node metastases; N+, with lymph node metastases; * *p* ≤ 0.05; ** *p* ≤ 0.01; *** *p* ≤ 0.001.

Expression of the *TCEA3* gene showed a similar trend in adenoma with early carcinoma and CRC N0 to both miRNAs, *miR-1225-3p* and *miR-1233-3p.* In adenoma and CRC N+, both miRNAs showed opposite trends in expression to its potential target gene *TCEA3*. The results are shown in Figure 10. We were not able to confirm any correlation between *TCEA3* and *miR-1225-3p* or *miR-1233-3p* (Table 4).

### 3.6. Gene and miRNA Correlation to the Level of Malignancy

The Spearman correlation coefficient showed that *L1TD1* and *SLITRK6* were significantly correlated to level of malignancy. *L1TD1* was weakly positively correlated, *SLITRK6* was moderately negatively correlated and *miR-1225-3p* and *miR-1233-3p* were significantly positively correlated to the level of malignancy (Table 5).

## 4. Discussion

We validated four genes related to CSC and CSC-like properties which were previously identified using bioinformatics analysis as differentially expressed between normal mucosa, adenoma and CRC [12]. We also validated miRNAs postulated by a bioinformatics approach as regulating these genes. We found that in CRC, expression of *ST6GALNAC1* decreased and expression of *L1TD1* increased with level of malignancy, whereas *SLITRK6* and *TCEA3* showed variable expression. *TCEA3* was also related to the malignant transformation of adenoma to adenoma with early carcinoma and the development of lymph node metastases in CRC. Furthermore, we found differential expression of miRNAs that potentially regulate these genes (*miR-199a-3p*, *miR-425-5p*, *miR-1225-3p*, *miR-1233-3p* and *miR-1303)* and a negative correlation between *miR-199a-3p* and its potential target gene *SLITRK6*. 

Expression of the *L1TD1* gene in our study progressively increased from adenoma to CRC, with the highest expression in CRC with lymph node metastases. L1TD1 has been shown to be associated with RNA binding, renewal of undifferentiated embryonal stem cells [13] and embryonal carcinoma cell lines [14]. In human embryonal stem cells, *L1TD1* has also been associated with canonical markers of pluripotency that are also involved in cancerogenesis, such as *OCT4*, *NANOG*, *LIN28* and *SOX2* [15]. With the use of bioinformatics analysis, a higher expression of *L1TD1* in CRC was shown to be associated with longer disease-free survival [16]. Our results showed a positive trend of expression of *L1TD1* to CRC cancerogenesis. However, its role remains speculative due to limited information on *L1TD1* in cancerogenesis. 

Our study showed variable expression of gene *SLITRK6* during CRC cancerogenesis. It was downregulated in all stages of CRC development, except in adenoma with early carcinoma, in which it was upregulated. *SLITRK6* has been shown to be highly expressed in neural stem and progenitor cells [17], and it has been associated with cytoskeletal dynamics, axon guidance and cell adhesion [18]. In other cancer types, it was expressed at high levels in bladder cancer and, to a lesser extent, in lung cancer, breast cancer and glioblastomas. Moreover, in bladder cancer, it was suggested as a promising target for conjugate therapy [19]. A bioinformatics study on CRC showed differentially expressed *SLITRK6* together with *L1TD1* and *ST6GALNAC1* [16], and it was downregulated in CRC compared to adenomas using microarray expression analysis [41]. However, our results showed no significant differences in expression between adenomas and CRC. This difference may be explained by the use of different methodologies for expression analysis of *SLITRK6* (microarrays versus qPCR).

Gene expression of *ST6GALNAC1* in our study progressively decreased from adenoma to CRC, with the lowest expression in CRC with lymph node metastases. This gene, and its product STn antigen, has been demonstrated to be associated with cell contact, cell migration and prognosis of patients with carcinoma of the colon, stomach, pancreas, breast, prostate and ovaries [23]. STn antigen has been used as a target in immunotherapy trials for breast, colon and ovarian cancer [23]. Data regarding its expression and function in normal human tissues are limited [16,23]. It has also been associated with stem cell maintenance in ovarian cancer [42], as well as with the maintenance of isolated stem cells of CRC [23]. Its upregulation has been associated with good prognosis in breast cancer [43] and enhanced tumorigenicity in a breast cancer cell line [44]. siRNA silencing of *ST6GALNAC1* led to reduced growth, migration and invasion of gastric cancer cells in vitro [45], whereas its overexpression enhanced their metastatic ability [46]. Due to limited data on its role in CRC and different patterns of expression in several cancers, further investigation is needed for better understanding the involvement of this gene in CRC cancerogenesis and metastatic spread. 

Gene *TCEA3* showed variable expression in our study, with significant upregulation in adenoma with early carcinoma and CRC without lymph node metastases. Interestingly, its expression was also significantly different between adenoma and CRC and between CRC without and with lymph node metastases, suggesting its role in metastases development. *TCEA3* was shown to have a higher expression level in mouse embryonal cells and was involved in regulation of stem cell differentiation [24]. Expression of *TCEA3* was lower in cell lines of ovarian carcinoma in which its interaction with receptor TGFβ I induced cell death [47]. *TCEA3* has also been associated with stomach cancer, in which high expression has been associated with better prognosis, lower proliferation of carcinoma cells and induction of apoptosis [48]. In a bioinformatics study of microarray expression data of normal colon tissue and CRC, *TCEA3* downregulation was identified among differentially expressed genes [49]. Our results are therefore consistent with previous findings on stomach cancer and CRC, thus contributing to understanding the involvement of *TCEA3* in CRC cancerogenesis.

When investigating correlations of genes/miRNAs with the level of malignancy, it is important to note that *SLITRK6* showed a moderate negative correlation and *L1TD1* was positively correlated with the level of malignancy. Among miRNAs, the expression of *miR-1225-3p* and *miR-1233-3p*, targeting *TCEA3*, were in weak positive correlation with the level of malignancy.

Interestingly, when investigating the miRNA-predicted target gene correlations, only *miR-199a-3p* and its target *SLITRK6* were in significant correlation. The pair was negatively correlated, which suggests inhibition of the target gene by the miRNA [50]. This correlation has not yet been previously observed in CRC.

*miR-199a-3p* was downregulated in our study in all investigated groups. However, no significant differences among the groups were found. In previous studies, *miR-199a-3p* was found to be highly expressed in the late stage of differentiation of human embryonal stem cells, as well as foetal pancreas and adult islet samples [51]. Additionally, *miR-199a-3p* was shown to target stemness and mitogenic-related pathways to suppress the expansion and tumorigenic capabilities of prostate cancer stem cells in vitro [52]. In CRC, *miR-199a-3p* was described as being significantly downregulated in the microarray expression data [53]. Upregulation of *miR-199a/b* contributed to cisplatin resistance in ALDHA1+ CRC stem cells [54]. Our data are consistent with previous microarray results on CRC. Further investigation of the exact involvement of *miR-199a-3p* in cancerogenesis of CRC is needed.

*miR-425-5p* was significantly upregulated in all investigated groups except CRC with lymph node metastases. Additionally, significant differences in expression were observed between adenoma and CRC and between CRC without and with lymph node metastases, suggesting its role in malignant transformation and the development of metastases. *miR-425-5p* has been previously associated with CRC, showing that *miR-425-5p* regulates chemoresistance in CRC cells [55] both in vitro and in vivo. A microarray analysis comparing isogenic chemo-sensitive and chemo-resistant HCT116 cell lines identified differentially expressed *miR-425-5p.* Xenograft mouse models showed that *miR-425-5p* inhibitor sensitized HCT116-R xenografts to chemotherapeutic drugs in vivo. *miR-425-5p* was also upregulated in a microarray expression experiment on CRC [56], and it was found that *miR-425-5p* downregulation impacted stemness and cisplatin resistance in laryngeal carcinoma cells [57]. Our results are consistent with previous microarray results on CRC.

*miR-1225-3p* is another miRNA in our study that was significantly upregulated in all investigated groups compared to normal mucosa. Additionally, it was also significantly differentially expressed between adenoma and CRC, suggesting a role in malignant transformation. Published data have shown that it was associated with the *TCEA3* gene in project GSE42095 performed on differentiated embryonic stem cells [51] and with CRC in project GSE35602 on CRC stromal tissue, in which it was upregulated [56]. Using microarray analysis, it was identified as one of the 173 differentially expressed miRNAs between spheroid body-forming cells (which possess gastric cancer stem cell properties) and parental cells on MKN-45 gastric cancer cell line cells [58]. Our results are consistent with microarray results on CRC stromal tissue.

*miR-1233-3p* was significantly upregulated in all investigated groups when compared to normal mucosa except CRC with lymph node metastases. Additionally, it was also significantly differentially expressed between adenoma and CRC, suggesting a role in malignant transformation. *miR-1233-3p* was associated with the *TCEA3* gene in project GSE28260, which was performed on renal cortex and medulla [59]. It was associated with CRC in a study performed on serum miRNA profiling in patients with colon adenomas or cancer, in which it was downregulated when comparing CRC to normal samples [60]. Before comparing our results to those performed on serum samples, it is important to note that, in addition to the fact that there are numerous differences in tissue types, there are also numerous differences in the methodologies used for profiling different tissue types.

*miR-1303* showed variable expression with significant upregulation in adenoma in comparison to normal mucosa. Additionally, it was also significantly differentially expressed between adenoma and CRC, suggesting a role in malignant transformation. *miR-1303* has been previously investigated in association with CRC, in which it was found to be part of a group with frequent and sometimes biallelic mutations in microsatellite instable (MSI) tumours. No direct link was found between the presence or absence of mono- or biallelic alterations and the levels of mature *miR-1303* expression in MSI cell lines. A significant increase in *miR-1303* was observed in microsatellite stable (MSS) CRC cell lines in comparison to normal colonic mucosa [61]. A correlation between *miR-1303* and *L1TD1* was also previously identified in the integrative knowledge base for miRNA-mRNA expression in colorectal cancer [28]. However, expression of this miRNA is variable, and there are limited data regarding its role in CRC cancerogenesis.

Genes associated with CSC features could be promising prognostic and therapeutic markers. It has been previously shown that CSC-associated molecular profiles can predict tumour regeneration and disease relapse after conventional therapy in CRC patients [9,62,63,64,65,66]. Direct targeting can be achieved by inhibiting self-renewal pathways, by interfering with antiapoptotic or metabolic pathways, by activating differentiation pathways or by acting on the protective microenvironment through the involved genes. Several potential anti-CSC targeted drugs have emerged in previous studies, with some of them making their way to the clinic [67]. As previously mentioned, *SLITRK6* is a promising candidate for conjugate therapy in bladder cancer [19] and the product of *ST6GALNAC1* has been a target in immunotherapy trials for several cancers [23]. Studying miRNAs regulating selected genes is also a promising therapeutic approach by silencing these genes using miRNAs mimic or by depleting miRNAs using antagomirs to re-express investigated genes [68].

One of the limitations of our study is related to normal samples, which were taken at least 20 cm away from the tumour and showed no microscopic abnormalities. However, genetic and protein aberrations may already be present in morphologically normal mucosa [69,70]. Despite certain limitations, these samples may be used as corresponding control samples to overcome differences in the genetic background. Additionally, the newly identified associations of these genes and miRNAs with CSCs, CRC development and progression in this study are of a preliminary nature. Further validation through a functional study may be needed for additional confirmation of the results. Another limitation is the relatively small sample size. The latter is due to the use of formalin-fixed paraffin-embedded (FFPE) tissue samples, in which nucleic acids are fragmented and therefore difficult to analyse. However, all FFPE cases were evaluated by pathologists, enabling appropriate diagnosis. Furthermore, only samples that successfully passed the initial quality control and samples with stable expression of the reference genes were selected for further analysis, thus limiting the number of included samples.

## 5. Conclusions

Using a bioinformatics approach, we identified and validated new CSC-related genes with a previously unknown or poorly defined role in CRC development and progression. Expression of three investigated genes progressively increased (*L1TD1)* or decreased *(ST6GALNAC1, SLITRK6)* with the level of malignancy. The *TCEA3* gene was also related to the malignant transformation of adenoma to adenoma with early carcinoma and development of lymph node metastases in CRC.

The expression of some of the potential regulatory miRNAs confirmed the alterations in gene expression in CRC development. Our results provide further evidence that CSC-related genes and their regulatory miRNAs are involved in CRC cancerogenesis and progression, and suggest that some of them, particularly *miR-199a-3p* and its *SLITRK6* target gene, are promising for further validation in CRC.

## Figures and Tables

**Table 1:** Selected probes.

Gene/miRNA	Assay ID	Sequence (Probe Sequence or Mature miRNA Sequence)
***B2M***	**Hs99999907_m1**	GTTAAGTGGGATCGAGACATGTAAG
***IPO8***	**Hs00183533_m1**	GGGGAATTGATCAGTGCATTCCACT
*L1TD1*	Hs00219458_m1	TTTTTCGCCAGGCACCAAGGCACAG
*SLITRK6*	Hs00536106_s1	TTTCCATGGACTGGAAAACCTGGAA
*ST6GALNAC1*	Hs01027885_m1	AGGAGGCCTTCAGACGACTTGCCCT
*TCEA3*	Hs00957468_m1	GAAATCGAAGATCATATCTACCAAG
*hsa-miR-199a-3p*	002304	ACAGUAGUCUGCACAUUGGUUA
*hsa-miR-335-5p*	000546	UCAAGAGCAAUAACGAAAAAUGU
*hsa-miR-425-5p*	001516	AAUGACACGAUCACUCCCGUUGA
*hsa-miR-1225-3p*	002766	UGAGCCCCUGUGCCGCCCCCAG
*hsa-miR-1233-3p*	002768	UGAGCCCUGUCCUCCCGCAG
***hsa-miR-1274b***	**002884**	UCCCUGUUCGGGCGCCA
*hsa-miR-1303*	002792	UUUAGAGACGGGGUCUUGCUCU
***RNU6B***	**001093**	CGCAAGGATGACACGCAAATTCGTGAAGCGTTCCATATTTTT

**Table 2:** Demographic characteristics of the included patients.

Patients	Adenoma	Adenoma with Early Carcinoma	CRC without Lymph Node Metastases	CRC with Lymph Node Metastases
M:F	10:1	9:4	4:6	9:4
Age	62.3 ± 10.7	64.9 ± 5.7	72.7 ± 11.6	73.2 ± 11.8

Legend: CRC, colorectal cancer; F, female; M, male.

**Table 3:** Condensed view of prioritization results for the miRNAs identified for further validation.

Gene	miRNA	Association with at Least Two Databases	Folding Free Energy Constraints	RNA22	Direct Validation
*L1TD1*	*hsa-miR-1303*	+	+	−	−
*SLITRK6*	*hsa-miR-199a-3p*	+	−	+	−
	*hsa-miR-425-5p*	−	+	−	+
*ST6GALNAC1*	*hsa-miR-335-5p*	+	+	−	−
*TCEA3*	*hsa-miR-335-5p*	+	+	−	−
	*hsa-miR-1225-3p*	+	+	−	−
	*hsa-miR-1233-3p*	+	+	+ *	−

Legend: CRC, colorectal carcinoma; *, did not appear as a significant binding pair, but had a folding energy higher than the software cut-off.

**Table 4:** Spearman’s correlation coefficients between expression of genes and their potentially regulatory miRNAs.

Gene and miRNA		Correlation Coefficient	Significance (2-Tailed)
*L1TD1*	*miR-1303*	−0.024	0.862
*SLITRK6*	*miR-425-5p*	−0.187	0.176
	*miR-199a-3p*	−0.323	0.017 *
*TCEA3*	*miR-1233-3p*	0.116	0.360
	*miR-1225-3p*	0.056	0.660

Legend: * *p* ≤ 0.05.

**Table 5:** Spearman correlation coefficients of the association between ΔCq of analysed genes and miRNAs and level of malignancy (from normal mucosa to adenoma, adenoma with early carcinoma, carcinoma without and carcinoma with lymph node metastases).

Gene and miRNA	Correlation Coefficient	Significance (2-Tailed)
***L1TD1***	0.336	0.011
***SLITRK6***	−0.433	<0.001
***ST6GALNAC1***	−0.186	0.141
***TCEA3***	0.102	0.419
***miR-199a-3p***	0.128	0.291
***miR-425-5p***	0.209	0.083
***miR-1225-3p***	0.345	0.003
***miR-1233-3p***	0.276	0.021
***miR-1303***	0.014	0.912

## Supplementary Materials

The following are available online at https://www.mdpi.com/2227-9059/9/2/179/s1.
